# Prognostic biomarkers for overall survival in patients undergoing surgery for bone metastases: a pan-cancer study

**DOI:** 10.3389/fmed.2025.1655245

**Published:** 2026-01-14

**Authors:** Elisa Belluzzi, Assunta Pozzuoli, Pietro Belloni, Maria Grazia Rodà, Andrea Angelini, Pietro Ruggieri

**Affiliations:** 1 Musculoskeletal Pathology and Oncology Laboratory, Department of Surgery, Oncology and Gastroenterology (DiSCOG), University of Padova, Padova, Italy; 2 Centre for Mechanics of Biological Materials, University of Padova, Padova, Italy; 3 Orthopedics and Orthopedic Oncology, Department of Surgery, Oncology and Gastroenterology (DiSCOG), University-Hospital of Padova, Padova, Italy; 4 Department of Statistical Sciences, University of Padova, Padova, Italy

**Keywords:** bone markers, bone metastasis, hematological markers, inflammatory markers, nomogram

## Abstract

Prompt diagnosis and prognostic assessment of bone metastases (BMs) remain challenging with most studies and prognostic models focusing on a single primary tumor and neglect host-related biomarkers. Therefore, this pan-cancer study aimed to evaluate bone metabolism, inflammatory, and hematological biomarkers in patients undergoing surgery for BMs, identify risk factors for BM development, and create a prognostic nomogram. A prospective cohort of adult patients with histologically confirmed BMs from various cancers was enrolled between 2020 and 2023. Baseline data included demographics, tumor type, and preoperative biomarkers such as bone turnover markers (P1NP, BAP), calcium, LDH, IGFBP-3, HALP, RDW, and NLR. Outcomes were recurrence, metastasis, and overall survival (OS), analyzed with Kaplan–Meier and Cox regression. Prognostic variables were integrated into a nomogram and validated by ROC curves and calibration. Ninety-one patients underwent surgery for BM. The most frequent primary cancers were breast (28.6%), kidney (23.1%), and lung (16.5%). Significant tumor-type differences were observed in BAP (*p* = 0.015), IGFBP-3 (*p* = 0.008), and HALP (*p* = 0.029). Univariate analysis identified P1NP, BAP, calcium, LDH, and RDW as prognostic markers. Multivariate models found age, kidney cancer, IGFBP-3, RDW, and NLR as independent predictors. The nomogram demonstrated strong predictive performance at 12 months (AUC = 82.3) and 24 months (AUC = 81.0). Tumor-specific and host-related biomarkers (IGFBP-3, NLR, and RDW) improved prognostic stratification beyond tumor type. The proposed nomogram demonstrated good discriminatory performance, supporting its potential use in personalized prognostic assessment and treatment planning.

## Introduction

The metastatic process involves the spread of tumor cells to distant sites, with bone being the third most common site after the lungs and liver (1, 2). Bone metastases (BMs) are gaining attention due to their significant healthcare, social, and financial impact (3, 4). The incidence of BMs is rising significantly due to advances in cancer treatments that enhance survival rates (5).

BM is characterized by disrupted bone homeostasis, driven by cancer cells, leading to osteolytic or osteoblastic lesions depending on the primary tumor type (6, 7).

BMs are common and result in severe complications, especially in breast, prostate and lung cancers, but they also occur in kidney and thyroid cancers (8). They commonly affect the spine, pelvis, and long bones, especially the femur (9). BMs often cause severe skeletal-related events (SREs), including fractures, spinal cord compression, hypercalcemia, and bone pain, reducing patients' quality of life and survival (5, 10).

BM is a complex process influenced by multiple factors and requiring a multidisciplinary approach (2, 4). The management involves timely diagnosis and both local and systemic treatments, including surgery, embolization, radiation therapy, chemotherapy, and bisphosphonates (11). Surgical resection of solitary metastasis or oligometastases (2 and 4 distant metastases in the same anatomic district) can improve prognosis in cancers like breast, kidney, prostate, bowel, thyroid cancers, and myeloma (9, 12). Key prognostic factors include the primary tumor type, visceral or brain metastases, pathological fractures, metastasis-free interval over 3 years, patient's general condition (e.g., hemoglobin levels), number of BMs, and previous chemotherapy (13).

Biomarkers of bone metabolism are essential for monitoring bone involvement in metastatic disease, as they provide insights into ongoing bone resorption and formation, which directly reflect the pathological remodeling processes associated with BM (7). Therefore, there is a considerable interest in exploring the prognostic and predictive value of these biomarkers.

Regarding bone resorption biomarkers, the β-isomerized C-terminal telopeptide of type beta-I collagen (β-CTX-I) is notable. Its elevation reflects increased degradation of type I collagen, indicating early adverse bone remodeling, particularly in breast and lung cancers (14–18). Markers of bone formation, such as total procollagen type 1 amino-terminal propeptide (P1NP), bone alkaline phosphatase (BAP) and osteocalcin are remarkable and associated with BM (18–21).

Calcium homeostasis is also critical in cancer-related bone pathology. Factors such as 1,25-dihydroxyvitamin D [1,25(OH)_2_D], parathyroid hormone (PTH), and calcium (Ca) influence bone metabolism in cancer, with low 1,25(OH)_2_D levels being associated with poorer outcomes in luminal breast cancer and non-small cell lung cancer (22–24). PTH contributes to BM progression by promoting bone resorption (6, 25).

Tumor-related inflammation significantly influences cancer progression and metastasis (26–28). Several inflammatory parameters including C-reactive protein (CRP), albumin, red blood cell distribution width (RDW), and ratios like neutrophil-to-lymphocyte (NLR) and platelet-to-lymphocyte (PLR), are widely recognized as prognostic markers in various cancers (29–35). Additionally, systemic scores like the systemic inflammatory index (SII), and the hemoglobin, lymphocyte, and platelet (HALP) score have shown prognostic value also in BM (29, 36–40).

These emerging biomarkers, in combination with other clinical factors, can play an essential role in preoperative survival estimation. By evaluating the full spectrum of prognostic indicators, including biomarkers, scoring systems, and patient-specific factors, surgeons can more accurately tailor surgical treatments to individual needs. For patients with a life expectancy longer than six months, more aggressive local treatments are often recommended (13).

This study aims to (i) evaluate bone metabolism, inflammatory and hematological biomarkers in patients undergoing surgery for BMs; (ii) identify risk factors for BM; and (iii) define a nomogram predicting survival of patients with BMs.

## Materials and methods

### Patients

In this prospective monocentric cohort study, consecutive patients with BMs from various malignancies, who were admitted to our institution for surgical treatment, were enrolled between 2020 and 2023. The inclusion criteria were as follows: (i) patients ≥18 years with BM from any primary malignancy, confirmed by histological analysis; (ii) patients able to provide informed consent to participate to the study. Exclusion criteria were as follows: (i) patients with primary bone tumors; (ii) patients with concomitant infectious diseases.

All patients gave their informed written consent after a detailed explanation of the risks and benefits. Institutional Ethics Committee approved this study (Ethic Committee Approval Code AOP2180) that was performed in accordance with the ethical standards of both the Declaration of Helsinki and the Good Clinical Practice.

### Patient assessment

Pre-operative clinical assessment of all patients included a complete clinical history, demographic [age, sex, body mass index (BMI), smoking habit, primary tumor, and comorbidities] and clinical features (bone metastatic site, number of BMs, presence of metastases in other site, and adjuvant treatments), and type of surgery were recorded. All patients were assessed radiographically on an outpatient basis with conventional radiographic examination (X-ray, MRI, and CT scans).

Patients were stratified according to the primary tumor as follows: breast, kidney, lung and other tumors (including prostate, uterine, urothelial, thyroid, Merkel cell neuroendocrine, salivary gland, and squamous cell carcinomas, adenoid cystic carcinoma of the mandible, leiomyosarcoma, lymphoma, melanoma, cholangiocarcinoma and pancreatic, bilio-pancreatic, and gastric adenocarcinomas, myxofibrosarcoma, and plasmacytoma).

### Laboratory methods

A fasting peripheral blood sample was collected early in the morning during the hospitalization period, prior surgery. Blood samples were processed within 30 min of collection to ensure accurate and consistent measurements. All analyses were performed at the Laboratory Medicine Unit of the University Hospital of Padova, accredited according to ISO 15189 standards. Internal and external quality controls are continuously monitored. The reference intervals values used in this study correspond to those established by the accredited laboratory and are detailed in Supplementary Table S1.

Standard hematological evaluation, including hemoglobin levels (HGB), Red Blood Cell Volume distribution width (RDW), white blood cell (WBC) count, neutrophil count, lymphocyte count, monocyte count and platelets count were analyzed using an automated blood count system, Sysmex (Siemens Healthcare GmbH ©).

P1NP, CTX, Osteocalcin, 1,25(OH)2D, and PTH levels were evaluated by using chemiluminescent immunoassay with the following assays: IDS-iSYS lntact P1NP and IDS-iSYS CTX-I (CrossLaps^®^) (Immunodiagnostic Systems, Boldon Colliery, United Kingdom), DiaSorin LIAISON^®^ Osteocalcin, DiaSorin LIAISON^®^ XL 1,25 Dihydroxyvitamin D, and DiaSorin LIAISON^®^ 1-84 PTH (DiaSorin Inc., Stillwater, Minnesota, USA).

BAP was measured using the IDS-iSYS Ostase^®^ BAP assay (Boldon Colliery, United Kingdom) through a spectrophotometric immunoenzymatic assay.

IGFBP-3 was evaluated by the DlAsource IGFBP-3-IRMA kit (DIAsource ImmunoAssays S.A., Louvain-la-Neuve, Belgium) based on an immunoradiometric assay.

LDH, Calcium and albumin were dosed by the Cobas c701 photometric analyzer with the following *in vitro* assays: LDHI2, Lactate Dehydrogenase ace. lo IFCC ver.2, CA2 Calcium Gen.2, and Tina-quant Albumin Gen.2, ALB-T TQ Gen.2 (Roche diagnostics, Basel, Switzerland).

β2-M was evaluated with the Kit β-2 Microglobulina Optilite^®^ (The Binding Site Group Ud, Birmingham, United Kingdom) based on a turbidimetric method.

CRP was evaluated by the CRP4, Tina-quant C-Reactive Protein IV kit based on an immunoturbidimetric assay (Roche diagnostics, Basel, Switzerland).

NLR, PLR, SII, SIRI, and HALP were calculated according to the following formula: NLR = neutrophils/lymphocyte; PLR = platelets/lymphocytes; SII = platelets × neutrophils /lymphocytes; SIRI = neutrophils × platelets/lymphocytes; and HALP = [HGB × albumin × lymphocytes]/platelets.

### Oncological outcomes

Oncological outcomes were evaluated based on the local recurrence, presence of metastasis, or death at last oncological follow-up. Patients were classified as follows: (i) no evidence of disease at the latest follow-up (NED); (ii) disease-free following treatment for local recurrence or metastasis (NEDrl or NEDm); (iii) alive with disease, with the presence of local recurrence or metastasis (AWD); and (iv) dead with disease (DWD). Survival was defined as the time from surgery to the last follow-up or death.

### Statistical analysis

Categorical variables (sex, smoking, HBP, hypothyroidism, dyslipidemia, diabetes, bone metabolism drugs, surgery, bone metastatic site, presence of other metastasis, presence of other tumors, chemotherapy, radiotherapy, AWD, DWD) were summarized with relative frequencies, while continuous variables (age, BMI, survival time, hematological and inflammatory markers) were summarized with mean and standard deviation. A Fisher's exact test was carried out to compare the frequency distribution of the categorical variables among tumor types, while an analysis of variance was used to evaluate the same distribution for the continuous variables. A multivariate Cox proportional hazards model was fitted to evaluate the impact of clinical variables (sex, age, tumor site (humerus, rachis, other), type of surgery (prosthesis, other), primary tumor type (kidney, lung, other), presence of non-BMs, other BMs, and time from diagnosis), bone metabolism markers (calcium, BAP, IGFBP-3, osteocalcin, CTX, P1NP, vitamin D, PTH), and inflammatory and hematological markers [albumin, LDH, β2-microglobulin, CRP, hemoglobin (HGB), RDW, neutrophils, NLR, platelets (PLT), platelet-to-lymphocyte ratio (PLR), SIRI, monocytes, WBC count, and HALP] on overall survival. The proportion of missing data was very small; therefore we decided not to proceed with any form of data imputation but to exclude the missing data from the model's design matrix. To facilitate the interpretability of the Cox models, markers (calcium, BAP, IGFB3, osteocalcin, CTX, P1NP, vitamin D, PTH, albumin, LDH, β2-M, CRP, HGB, RDW, neutrophils, PLT, monocytes, WBC, non-bone metastasis, and other BM) were dichotomized (in range vs out of range). To further help healthcare personnel to use the information carried by biomarkers data, we fitted a reduced version of the Cox model, selecting the variables with a bi-directional stepwise procedure base on the Akaike information criterion (AIC). This reduced model was depicted in a nomogram, that is a visual representation of the model. In a nomogram, we can represent each covariate with its own scale, and each scale translates the value of its covariate into a certain number of points. These points reflect how much that covariate contributes to the final prediction. Summing the point values for all covariates provides the predicted survival probability. Therefore, the nomogram turns the regression model into a visual system where predictors are displayed as linear scales, points quantify their effects and the sum of points correspond to the final prediction. A cut-off of 0.05 for *p*-values was chosen to indicate statistically significant findings. The statistical analysis was performed using the statistical software R (ver. 4.4.2) (41).

## Results

### Patients

During a 4-year period, a total of 91 patients with BM were surgically treated at our institution. Demographic and clinical features of the entire cohort and categorized by tumor type at baseline are reported in Table 1. Females are significantly more represented (64.8 %, *p* < 0.001). Primary tumors were most commonly located in the breast (28.6%), kidney (23.1%), and lung (16.5%), with other sites accounting for 31.9%.

**Table 1:** Socio-demographic and clinical features of the overall cohort and stratified by tumor type at baseline.

**Parameter**	**Overall cohort (*n* = 91)**	**Breast (*n* = 26)**	**Kidney (*n* = 21)**	**Lung (*n* = 15)**	**Other (*n* = 29)**	***P*-value**
Age, years	66.2 ± 12.2	62.2 ± 10.8	70.5 ± 10.9	68.1 ± 8.4	65.7 ± 14.8	0.117
**Sex**, ***n*** **(%)**						**<0.001**
Male	32 (35.2)	0 (0)	12 (57.1)	5 (33.3)	15 (51.7)	
Female	59 (64.8)	26 (100.0)	9 (42.9)	10 (66.7)	14 (48.3)	
BMI, kg/m^2^	25.1 ± 4.1	25.4 ± 4.4	24.5 ± 4.2	26.0 ± 4.7	24.6 ± 3.6	0.680
**Smoking**, ***n*** **(%)**						0.072
No	49 (53.8)	19 (73.1)	10 (47.6)	4 (26.7)	16 (55.2)	
Yes	9 (9.9)	0 (0)	3 (14.3)	3 (20)	3 (10.3)	
Former	33 (36.3)	7 (26.9)	8 (38.1)	8 (53.3)	10 (34.5)	
**HBP**, ***n*** **(%)**						**0.028**
No	55 (60.4)	19 (73.1)	7 (33.3)	9 (60)	20 (69)	
Yes	36 (39.6)	7 (26.9)	14 (66.7)	6 (40)	9 (31)	
**Hypothyroidism**, ***n*** **(%)**						0.386
No	77 (84.6)	24 (92.3)	17 (81)	11 (73.3)	25 (86.2)	
Yes	14 (15.4)	2 (7.7)	4 (19)	4 (26.7)	4 (13.8)	
**Dyslipidemia**, ***n*** **(%)**						0.283
No	72 (79.1)	23 (88.5)	14 (66.7)	11 (73.3)	24 (82.8)	
Yes	19 (20.9)	3 (11.5)	7 (33.3)	4 (26.7)	5 (17.2)	
**Diabetes**, ***n*** **(%)**						0.051
No	82 (90.1)	25 (96.2)	18 (85.7)	11 (73.3)	28 (96.6)	
Yes	9 (9.9)	1 (3.8)	3 (14.3)	4 (26.7)	1 (3.4)	
**Bone metabolism drugs**, ***n*** **(%)**						**0.009**
No	38 (41.8)	4 (15.4)	11 (52.4)	7 (46.7)	17 (58.6)	
Yes	53 (58.2)	22 (84.6)	10 (47.6)	8 (53.3)	12 (41.4)	

BMI, body mass index; HBP, high blood pressure. The data are reported as mean ± standard deviation or number and percentage. *P*-values refer to a Fisher's exact test for count data for comparison between discrete variables and to an analysis of variance for comparison between a discrete variable and a continuous variable. Values with *p* < 0.05 are shown in bold.

Significant differences among the various groups of patients were highlighted only for sex (*p* < 0.001), blood pressure (*p* = 0.028), and the use of drugs impacting bone metabolism (*p* = 0.009).

### Surgical and clinical (oncological) features

Surgical and baseline oncological features of the overall cohort, as well by tumor type, are presented in Table 2. The femur was the most commonly involved site of metastasis across all tumor types, with most patients treated using endoprosthetic replacement, followed by intramedullary nailing. Local and systemic therapies were administered to more than half of the patients. A difference between the groups were observed regarding treatment with chemotherapy (*p* = 0.001), radiotherapy (*p* = 0.023) and survival time (*p* = 0.006). The longest survival time was observed in patients with kidney cancer (17.3 ± 11.0 months), while the shortest in patients with lung cancer (7.0 ± 7.0 months).

**Table 2:** Surgical and oncological features of the overall cohort and stratified by tumor type.

**Parameter**	**Overall cohort (*n* = 91)**	**Breast (*n* = 26)**	**Kidney (*n* = 21)**	**Lung (*n* = 15)**	**Other (*n* = 29)**	***P*-value**
**Surgery**, ***n*** **(%)**						0.155
Nail	28 (29.4)	6 (23.1)	5 (23.8)	8 (53.3)	9 (31)	
Prosthesis	44 (48.4)	15 (57.7)	8 (38.1)	5 (33.3)	16 (55.2)	
Other	19 (20.9)	5 (19.2)	8 (38.1)	2 (13.3)	4 (13.8)	
**Bone metastatic site**, ***n*** **(%)**						0.095
Femur	57 (62.6)	21 (80.8)	8 (38.1)	9 (60)	19 (65.5)	
Humerus	21 (23.1)	4 (15.4)	6 (28.6)	4 (26.7)	7 (24.1)	
Rachis	6 (6.6)	1 (3.8)	2 (9.5)	1 (6.7)	2 (6.9)	
Other	7 (7.7)	0 (0)	5 (23.8)	1 (6.7)	1 (3.4)	
**Other bone metastases**, ***n*** **(%)**						**0.025**
No	28 (30.8)	4 (15.4)	10 (47.6)	2 (13.3)	12 (41.4)	
Yes	63 (69.2)	22 (84.6)	11 (52.4)	13 (86.7)	17 (58.6)	
**Other sites of metastasis**, ***n*** **(%)**						0.954
No	42 (46.1)	12 (46.1)	9 (42.9)	8 (53.3)	13 (44.8)	
Yes	49 (53.9)	14 (53.9)	12 (57.1)	7 (46.7)	16 (55.2)	
**Other tumor**, ***n*** **(%)**						0.672
No	76 (83.5)	22 (84.6)	17 (81)	14 (93.3)	23 (79.3)	
Yes	15 (16.5)	4 (15.4)	4 (19)	1 (6.7)	6 (20.7)	
**Chemotherapy**, ***n*** **(%)**						**0.001**
No	33 (36.3)	2 (7.7)	12 (57.1)	4 (26.7)	15 (51.7)	
Yes	58 (63.7)	24 (92.3)	9 (42.9)	11 (73.3)	14 (48.3)	
**Radiotherapy**, ***n*** **(%)**						**0.023**
No	41 (45.1)	5 (19.2)	12 (57.1)	8 (53.3)	16 (55.2)	
Yes	50 (54.9)	21 (80.8)	9 (42.9)	7 (46.7)	13 (44.8)	
Survival time, months	11.2 ± 10.3	10.7 ± 10.3	17.3 ± 11.0	7.0 ± 7.0	9.6 ± 9.6	**0.006**
**Status**, ***n*** **(%)**						0.051
AWD	56 (61.5)	13 (50.0)	16 (76.2)	6 (40)	21 (72.4)	
DWD	35 (38.5)	13 (50.0)	5 (23.8)	9 (60)	8 (27.6)	

AWD, alive with disease; DWD, died with disease. The data are reported as mean ± standard deviation or number and percentage. *P*-values refer to Fisher's exact test for count data. Values with *p* < 0.05 are shown in bold.

### Bone metabolism, hematological and inflammatory markers

Bone metabolism and other biomarkers for the entire cohort and by tumor type are reported in Table 3. Significant differences were observed for BAP (*p* = 0.015) with higher values in breast cancer (approximately half of the patients had normal values) and the lowest values in kidney cancer (over 90% had normal values). IGFBP-3 also showed significant differences, with breast cancer being associated with more out-of-range values (42.3 % of the patients) (*p* = 0.008).

**Table 3:** Biomarker levels in the overall cohort and stratified by tumor type.

**Marker**	**Overall cohort (*n* = 91)**	**Breast (*n* = 26)**	**Kidney (*n* = 21)**	**Lung (*n* = 15)**	**Other (*n* = 29)**	***P*-value**
**Beta-CTX-I, pg/mL**						
Mean ± SD	736.9 ± 961.6	1013.7 ± 1620.2	711.6 ± 573.7	561.1 ± 355.9	598 ± 471	0.104
In range, *n* (%)	76 (83.5)	19 (73.1)	20 (95.2)	11 (73.3)	26 (89.7)	
Out of range, *n* (%)	15 (16.5)	7 (26.9)	1 (4.8)	4 (26.7)	3 (10.3)	
**P1NP**, μ**g/L**						
Mean ± SD	94.5 ± 131.9	172.5 ± 215.8	39.4 ± 15	80.3 ± 90.3	71.8 ± 39.2	0.647
In range, *n* (%)	65 (71.4)	16 (61.5)	16 (76.2)	11 (73.3)	22 (75.9)	
Out of range, *n* (%)	26 (28.6)	10 (38.5)	5 (23.8)	4 (26.7)	7 (24.1)	
**BAP**, μ**g/L**						
Mean ± SD	25.9 ± 23.4	35.1 ± 29.4	16.8 ± 9.0	24.9 ± 28.5	24.7 ± 19.5	**0.015**
In range, *n* (%)	70 (76.9)	14 (53.8)	19 (90.5)	13 (86.7)	24 (82.8)	
Out of range, *n* (%)	21 (23.1)	12 (46.2)	2 (9.5)	2 (13.3)	5 (17.2)	
**Osteocalcin**, μ**g/L**						
Mean ± SD	16.4 ± 12.2	19.1 ± 16.9	17.2 ± 7.3	12.5 ± 9.3	15.3 ± 11.3	0.459
In range, *n* (%)	78 (85.7)	22 (84.6)	19 (90.5)	11 (73.3)	24 (88.9)	
Out of range, *n* (%)	13 (14.3)	4 (15.4)	2 (9.5)	4 (26.7)	3 (11.1)	
**1,25(OH)**_2_ **D, pmol/L**						
Mean ± SD	99.2 ± 80.4	126.1 ± 101.9	73.2 ± 34.8	122 ± 110.2	81.7 ± 51.8	0.470
In range, *n* (%)	66 (72.5)	17 (65.4)	18 (85.7)	11 (73.3)	20 (69)	
Out of range, *n* (%)	24 (26.4)	9 (34.6)	3 (14.3)	4 (26.7)	8 (27.6)	
Missing data					1 (3.4)	
**PTH, ng/L**						
Mean ± SD	23.5 ± 18.8	23.7 ± 22.4	27 ± 20.7	24.9 ± 19.9	20 ± 12.6	0.679
In range, *n* (%)	60 (65.9)	17 (65.4)	14 (66.7)	8 (53.3)	21 (72.4)	
Out of range, *n* (%)	31 (34.1)	9 (34.6)	7 (33.3)	7 (46.7)	8 (27.6)	
**Calcium, mmol/L**						
Mean ± SD	2.3 ± 0.2	2.3 ± 0.2	2.4 ± 0.2	2.3 ± 0.3	2.3 ± 0.2	0.116
In range, *n* (%)	72 (79.1)	21 (80.8)	20 (90.5)	10 (66.7)	21 (72.4)	
Out of range, *n* (%)	19 (20.9)	5 (19.2)	1 (4.8)	5 (33.3)	8 (27.6)	
**IGFBP-3**, μ**g/L**						
Mean ± SD	3057.4 ± 1174.3	3502 ± 1123.8	2976.2 ± 947.8	3082.9 ± 1096.2	2678.4 ± 1239.7	**0.008**
In range, *n* (%)	64 (70.3)	15 (57.7)	17 (81)	15 (100)	17 (58.6)	
Out of range, *n* (%)	25 (27.5)	11 (42.3)	4 (19)	0 (0)	10 (34.5)	
Missing data					2 (6.9)	
**LDH, U/L**						
Mean ± SD	299.9 ± 277.8	320.2 ± 369.4	222.9 ± 76.4	325.5 ± 183.2	325 ± 316.7	0.301
In range, *n* (%)	33 (36.3)	8 (30.8)	10 (47.6)	3 (20)	12 (41.4)	
Out of range, *n* (%)	57 (62.6)	18 (69.2)	11 (52.4)	12 (80)	16 (55.2)	
Missing data					1 (3.4)	
β**2-M, mg/L**						
Mean ± SD	2.7 ± 1.6	2.4 ± 1.2	3.1 ± 1.1	2.6 ± 1.0	2.8 ± 2.3	0.053
In range, *n* (%)	48 (52.7)	18 (69.2)	7 (33.3)	6 (40)	17 (58.6)	
Out of range, *n* (%)	42 (46.2)	8 (30.8)	14 (66.7)	9 (60)	11 (37.9)	
Missing data					1 (3.5)	
**CRP, mg/L**						
Mean ± SD	56.1 ± 64.5	44.6 ± 53.9	33.4 ± 42.0	93.1 ± 75.9	63.8 ± 73.1	0.294
In range, *n* (%)	19 (20.9)	8 (30.8)	5 (23.8)	1 (6.7)	5 (17.2)	
Out of range, *n* (%)	72 (79.1)	18 (69.2)	16 (76.2)	14 (93.3)	24 (82.8)	
**Albumin, g/L**						
Mean ± SD	30.1 ± 7.8	31.7 ± 8.4	33.2 ± 6.5	25.9 ± 6.0	28.6 ± 8.0	0.228
In range, *n* (%)	29 (31.9)	11 (42.3)	8 (38.1)	2 (13.3)	8 (27.6)	
Out of range, *n* (%)	62 (68.1)	15 (57.7)	13 (61.9)	13 (86.7)	21 (72.4)	

Beta-CTX-I, beta-isomerized C-terminal telopeptide of type I collagen; P1NP, total procollagen type 1 amino-terminal propeptide; BAP, bone alkaline phosphatase; 1,25(OH)2D, 1,25-dihydroxyvitamin D; PTH, parathyroid hormone; IGFBP-3, insulin-like growth factor binding protein-3; LDH, lactate DeHydrogenase; β2-M, beta 2 microglobulin; CRP, C-reactive protein. Data are reported as mean ± standard deviation or number and percentage. *P*-values refer to Fisher's exact test for count data. Values with *p* < 0.05 are shown in bold.

Hematological, inflammatory markers and related scores are described in Table 4. Significant differences were observed only for HALP (*p* = 0.029) with higher values in patients with kidney cancer.

**Table 4:** Hematological and inflammatory markers and related scores in the overall cohort and by tumor type.

**Marker**	**Overall cohort (*n* = 90)**	**Breast (*n* = 26)**	**Kidney (*n* = 20)**	**Lung (*n* = 15)**	**Other (*n* = 29)**	***P*-value**
**HGB, g/L**						
Mean ± SD	108.6 ± 14.2	106.5 ± 14.6	115.3 ± 15.0	107.7 ± 12.2	106.4 ± 13.4	0.752
In range, *n* (%)	9 (10)	2 (7.7)	3 (14.3)	2 (13.3)	2 (6.9)	
Out of range, *n* (%)	81 (90)	24 (92.3)	17 (81)	13 (86.7)	27 (93.1)	
**RDW**						
Mean ± SD	15.5 ± 2.7	16.1 ± 2.4	6.6 ± 2.0	15.0 ± 2.0	16.0 ± 3.4	0.201
In range, *n* (%)	56 (62.2)	13 (50)	15 (75)	10 (66.7)	17 (58.6)	
Out of range, *n* (%)	34 (37.8)	13 (50)	5 (25)	5 (33.3)	12 (41.4)	
**Neutrophils, 10**^9^ **g/L**						
Mean ± SD	5.7 ± 2.6	4.6 ± 2.4	6.6 ± 2.0	6.4 ± 3.2	5.6 ± 2.6	0.797
In range, *n* (%)	67 (74.4)	21 (80.8)	15 (75)	11 (73.3)	20 (69)	
Out of range, *n* (%)	23 (25.6)	5 (19.2)	5 (5)	4 (26.7)	9 (31)	
**NLR**						
Mean ± SD	5.8 ± 7.0	6.6 ± 11.3	5.9 ± 4.5	5.9 ± 3.3	5.1 ± 4.8	0.878
**PLT, 10**^9^ **g/L**						
Mean ± SD	293.4 ± 101.4	303 ± 110.5	268.6 ± 83.0	304.6 ± 99.6	296.1 ± 107.4	0.414
In range, *n* (%)	75 (83.3)	20 (76.9)	19 (95)	12 (80)	24 (82.8)	
Out of range, *n* (%)	15 (16.7)	6 (23.1)	1 (5)	3 (20)	5 (17.2)	
**PLR**						
Mean ± SD	272.9 ± 157.6	336.3 ± 200.5	231.1 ± 148.0	304.6 ± 99.6	242.2 ± 124.0	0.079
**SII**						
Mean ± SD	1655.6 ± 1769.9	1806.1 ± 2522.8	1664.1 ± 1710.5	1774 ± 1070.6	1453.4 ± 1282.4	0.892
**SIRI**						
Mean ± SD	3.8 ± 3.3	3.2 ± 3.3	3.9 ± 2.1	5.1 ± 4.3	3.7 ± 3.5	0.338
**Monocytes, 10**^9^ **g/L**						
Mean ± SD	0.7 ± 0.3	0.6 ± 0.2	0.7 ± 0.2	0.8 ± 0.3	0.7 ± 0.3	0.317
In range, *n* (%)	74 (82.2)	24 (92.3)	17 (85)	11 (73.3)	22 (75.9)	
Out of range, *n* (%)	16 (17.8)	2 (7.7)	3 (15)	4 (26.7)	7 (24.1)	
**WBC, 10**^9^ **g/L**						
Mean ± SD	7.9 ± 2.9	6.4 ± 2.4	8.7 ± 2.1	8.7 ± 3.8	8.1 ± 3.0	0.445
In range, *n* (%)	68 (75.6)	21 (80.8)	17 (85)	10 (66.7)	20 (69)	
Out of range, *n* (%)	22 (24.4)	5 (19.2)	3 (15)	5 (33.3)	9 (31)	
**HALP**						
Mean ± SD	16.6 ± 11.4	14.0 ± 9.3	22.1 ± 12.1	11.8 ± 5.6	17.6 ± 13.5	**0.029**

HGB, hemoglobin; RDW, red blood cell volume distribution width; NLR, neutrophil-lymphocyte ratio; PLT, platelet; PLR, platelet-lymphocyte ratio; SII, systemic inflammatory index; SIRI, systemic inflammation response index; WBC, white blood cells; HALP, hemoglobin, albumin, lymphocyte, and platelet counts (HALP). *P*-values refer to a Fisher's exact test for count data for comparison between discrete variables and to an analysis of variance for comparison between a discrete variable and a continuous variable. Values with *p* < 0.05 are shown in bold.

### Prognostic factors

**Figure 1:**
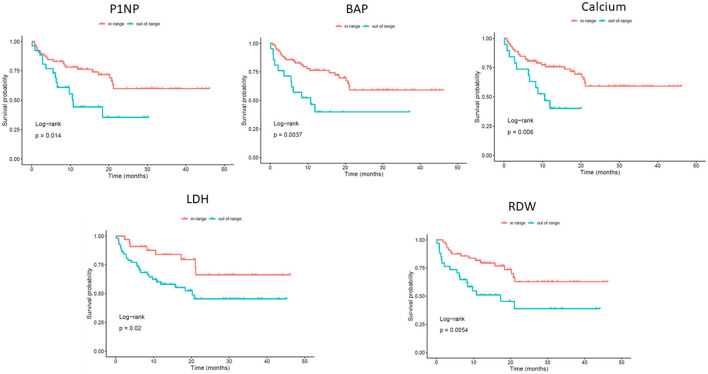
Kaplan–Meier survival curves showing 3-years survival probability according to selected biomarkers. Patients were stratified into “in-range” vs. “out-of-range” groups for each biomarker: P1NP (*p* = 0.014); BAP (*p* = 0.0037); Calcium (*p* = 0.006); LDH (*p* = 0.02); RDW (*p* = 0.0054). *P*-values are derived from log-rank tests comparing survival distributions between groups.

Log-rank analysis for specific survival after metastatic occurrence identified several significant prognostic factors, including P1NP (*p* = 0.014), BAP (*p* = 0.0037), calcium (*p* = 0.006), LDH (*p* = 0.02), and RDW (*p* = 0.0054) (Figure 1).

Multivariate Cox proportional hazards models were carried out. Age (*p* = 0.001), IGFBF3 (*p* = 0.041), RDW (*p* = 0.024), NLR (*p* = 0.037) were found to be significant predictors. Calcium (*p* = 0.051) was observed to be borderline (Table 5). Only kidney primary tumor was significantly associated with better prognosis (HR = 0.08, *p* = 0.017) compared to breast cancer (reference category).

**Table 5:** Multivariate Cox proportional hazards models.

**Predictors**	**Harzard ratio**	**CI**	***P*-value**
Sex [male]	1.44	0.32–6.43	0.631
Age	1.14	1.05–1.24	**0.001**
Site [humerus]	1.53	0.30–7.73	0.610
Site [rachis]	1.71	0.03–112.01	0.802
Site [other]	45.11	0.90–2120.82	0.153
Surgery [prosthesis]	0.30	0.08–1.14	0.177
Surgery [other]	0.07	0.00–1.45	0.386
Primary tumor [kidney]	0.08	0.01–0.64	**0.017**
Primary tumor [lung]	0.49	0.09–2.67	0.408
Primary tumor [other]	0.23	0.04–1.22	0.084
Calcium (0 = in range)	3.73	0.73–19.13	0.051
BAP (0 = in range)	2.67	0.52–13.66	0.239
IGFBF-3 (0 = in range)	2.98	1.70–12.75	**0.041**
Osteocalcin (0 = in range)	0.98	0.17–5.53	0.979
CTX (0 = in range)	0.87	0.14–5.46	0.884
P1NP (0 = in range)	2.36	0.64–8.76	0.199
Vitamin D (0 = in range)	0.22	0.06–1.81	0.124
PTH (0 = in range)	0.99	0.21–4.76	0.989
Albumin (0 = in range)	3.93	0.49–31.37	0.196
LDH (0 = in range)	4.01	0.95–16.98	0.159
β2-M (0 = in range)	0.16	0.03–1.97	0.147
CRP (0 = in range)	1.29	0.26–11.74	0.756
HGB (0 = in range)	1.79	0.27–11.74	0.545
RDW (0 = in range)	2.14	0.62–0.81	**0.024**
Neutrophils (0 = in range)	0.99	0.16–6.11	0.988
NLR	1.05	1.86–2.14	**0.037**
PLT (0 = in range)	3.75	0.99–14.11	0.061
PLR	1.00	0.99–1.01	0.847
SIRI	0.85	0.66–1.10	0.224
Monocytes (0 = in range)	3.34	0.46–24.02	0.231
WBC (0 = in range)	1.62	0.29–9.11	0.587
HALP	0.98	0.82–1.16	0.784
Non-bone metastasis (0 = no)	2.59	0.77–8.70	0.124
Other bone metastasis (0 = no)	1.33	0.20–8.69	0.764
Time from diagnosis	0.92	0.79–1.07	0.276
Observations	52		
*R* ^2^	0.566		

BAP, bone alkaline phosphatase; IGFBP-3, insulin-like growth factor binding protein-3; Beta-CTX-I, beta-isomerized C-terminal telopeptide of type I collagen; P1NP, total procollagen type 1 amino-terminal propeptide; 1,25(OH)2D, 1,25-dihydroxyvitamin D; PTH, parathyroid hormone; LDH, lactate DeHydrogenase; β2-M, beta 2 microglobulin; CRP, C-reactive protein; HGB, hemoglobin; RDW, red blood cell volume distribution width; NLR, neutrophil-lymphocyte ratio; PLT, platelet; PLR, platelet-lymphocyte ratio; SIRI, systemic inflammation response index; WBC, white blood cells; HALP, hemoglobin, albumin, lymphocyte, and platelet counts (HALP). Values with *p* < 0.05 are shown in bold.

When focusing specifically on bone markers, the Cox model showed that age (*p* = 0.042), and BAP (*p* = 0.019) were found significantly associated with poor survival (Supplementary Table S2). Considering inflammatory and hematological markers, age (*p* = 0.003), kidney as primary tumor (*p* = 0.028), and RDW (*p* = 0.035) were statistically significant (Supplementary Table S3).

### Construction and validation of a prognostic nomogram

**Figure 2:**
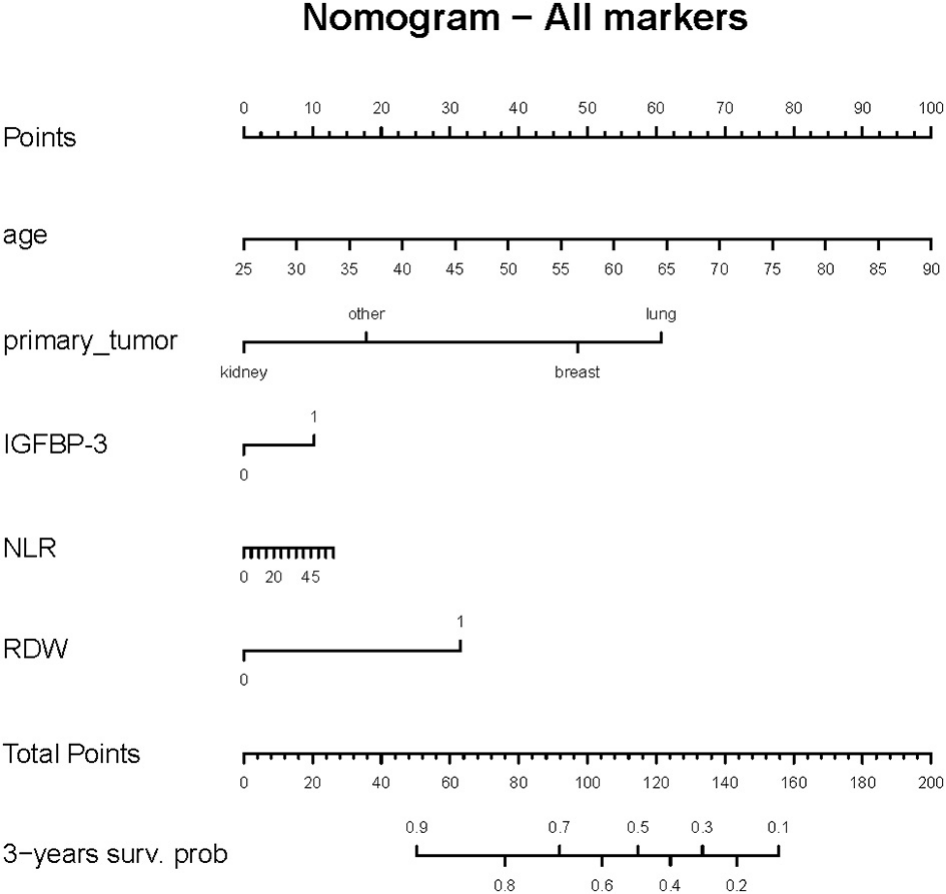
Nomogram predicting 3-year survival probability for the overall cohort. Each predictor variable (age, primary tumor type, IGFBP-3, RDW and NLR) is assigned a score on the top scale. By summing the scores for all variables, a total score is obtained, which corresponds to the predicted 3-year survival probability shown on the bottom axis.

A nomogram was developed based on prognostic risk factors with an associate *p*-value less than 0.05, observed in the model that included all the analyzed markers (Figure 2).

**Figure 3:**
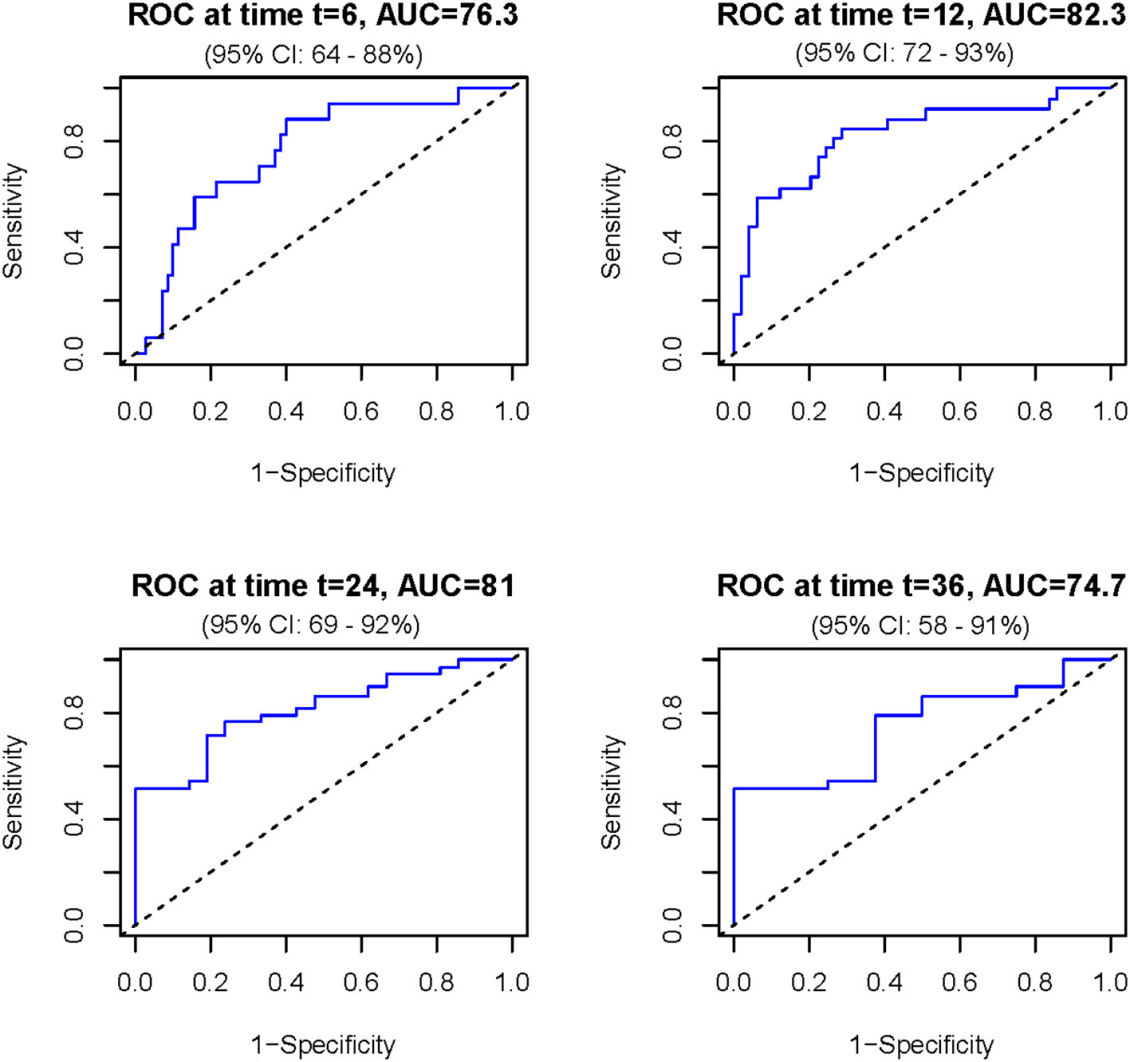
Receiver operating characteristic (ROC) curves assessing the discriminatory ability of the prognostic model for overall survival at different time points. The model showed consistently moderate to good accuracy (between 76.3 and 82.3), with the highest area under the curve (AUC = 82.3) observed between 12 and 24 months. AUC is expressed in percentage and time in months.

Then, the nomogram was validated and results indicated that the Cox model had a good predictive ability for survival over time. Specifically, the ROC curves showed that the model's predictive performance varies across time points (Figure 3). The discriminative ability is good between 12 and 24 months, with the highest AUC observed at 12 months (AUC = 82.3 %), meaning the model is effective in distinguishing between surviving and deceased patients at this timeframe. At other time points, such as 6 and 36 months, the performance is moderate.

## Discussion

Prompt and early diagnosis of BM remains challenging due to the slow radiological changes that occur with disease progression, as well as the similarity of BM to many other non-malignant skeletal diseases (42, 43). Therefore, the identification of biomarkers for early diagnosis, prognosis, therapeutic monitoring, and targeting could enhance the clinical management of patients with BMs, thereby improving their prognosis and quality of life of patients. Most studies published to date have focused on BMs originating from a single primary tumor type (e.g., breast, lung, prostate). Our study, however, adopts a pan-cancer approach, including patients with BM from a range of primary cancers. This allows us to investigate BM across different tumor types, providing insights that single-tumor studies may not capture.

Significant differences were observed among primary tumor types for various biomarkers, particularly BAP, IGFBP-3, and HALP. Notably, higher BAP levels were found in BM patients with breast cancer. A recent systematic review with meta-analysis highlighted that breast cancer patients with BMs had higher serum levels of BAP and shorter survival time compared to non-metastatic patients (44). BAP is the specific bone-isoform of alkaline phosphatase produced by osteoblasts during bone formation (45). BAP was found to be a good diagnostic factor for metastasis in patients with breast cancer and lung cancer (44, 46, 47). Moreover, BAP was identified as a prognostic factor in a meta-analysis in BM patients with breast cancer (48). IGFBP-3 is the most multifunctional IGFBP studied and the most abundant in the circulation. It regulates the activity of IGF-1, which is crucial for bone remodeling and tumor growth in metastatic sites. IGFBP-3 can act as a modulator, either promoting or inhibiting metastasis depending on the tumor microenvironment (49). In our study, higher levels of this protein were found in BM patients with breast cancer compared to patients with BM from other cancers. The HALP score, which reflects the nutritional and inflammatory status of patients, was found to be highest in BM patients with kidney cancer and lowest in BM with lung cancer.

Log-rank analysis identified several factors associated with overall survival, including P1NP, BAP, calcium, LDH, and RDW. Notably, patients with out-of-range values for these markers showed significantly shorter survival compared to those with values within the normal range. Serum P1NP levels reflect the rate of new bone formation, as it is released into the bloodstream during the synthesis of type I collagen, which is the main collagen found in bone. In literature, elevated levels of P1NP were found to be higher in patients with BM in different types of cancer (50, 51) and to be a good diagnostic tool for predicting patients with BM (52–54). Moreover, high levels of P1NP were linked with a short time to development of BM and low overall survival overall survival in patients with breast cancer (52, 53). In our study, we found that patients with abnormal calcium levels had lower survival rates. It should be empathized that, in our cohort, most of these patients presented with hypocalcemia (12 out 19). While symptomatic hypocalcemia is typical associated with osteoblastic BM (55), its presence in patients with osteolytic BM suggests a more complex interaction with bone metabolism. In osteolytic metastasis, bone destruction usually leads to elevated calcium levels, but our findings suggest that compensatory mechanisms may be at play, potentially involving bone resorption-related pathways. In literature, lower blood calcium levels were found to be associated with a higher risk of unfavorable prognosis and BM of non-small cell lung cancer (56). Additionally, calcium was also found to be a risk factor for bone metastasis in renal cell carcinoma (57). In our multivariate model, calcium was found to be borderline.

LDH is a glycolytic enzyme, which is released into the extracellular environment from cells in case of membrane damage. LDH serum concentrations increase following tissue injury or during disease states. In the context of cancer, it is considered a non-specific but valuable marker and high levels suggest high tumor burden, rapid cell turnover or tissue damage. In accordance with our study, high levels of LDH have been reported to be negative prognostic factors for BM in patients with lung cancer and breast cancer (58, 59). A systematic review with meta-analysis showed that elevated LDH levels were associated with poor overall survival and progression-free survival in patients with metastatic renal cancer (60).

RDW is a measurement of variability and size of red blood cells with higher values indicating greater heterogeneity in cell sizes, typically linked with chronic inflammatory diseases (61, 62). In our study, we found that BM patients with elevated RDW had an overall poor survival and it was found to be an independent significant factor impacting overall survival of the patients in multivariate cox regression. In literature, RDW was reported as an independent prognostic factor of poor outcomes in different cancer patients, including breast cancer and lung cancer (63–66). Higher levels were associated with increased mortality rates in metastatic renal cell carcinoma (67).

Multivariate Cox models confirmed the independent prognostic role of age, IGFBP-3, RDW and NLR. Importantly, kidney as primary tumor was found to be protective in the Cox model. The association between age and worse prognosis in BM aligns with several previous studies (68–70). Older patients are more likely to have comorbidities' such as diabetes, cardiovascular diseases, which complicate cancer treatment and negatively affect the overall health (71). Moreover, age-related frailty and a decline in physiological reserve often reduce the tolerance to aggressive treatments like chemotherapy, radiotherapy, or surgical interventions (72). Although lung cancer shows the poorest overall survival in metastatic settings (4.5%), renal cell carcinoma demonstrates one of the most dramatic declines in prognosis when distant spread occurs (12.3%) (73). The substantial drop in 5-year survival, coupled with a relatively high incidence of BM and frequent need for surgical intervention, underscores the clinical aggressiveness of BM kidney cancer. Moreover, it has been reported that metastatic lesions can occur at late follow-up in kidney cancer patients (74).

In our study only NLR, a marker of systemic inflammation and immune status, was identified to be an independent prognostic predictor. High NLR reflects a shift toward a protumor inflammatory milieu—driven by tumor-derived cytokines that stimulate neutrophil proliferation and activation—and concurrent lymphopenia resulting from stress-induced corticosteroid release and tumor-mediated immunosuppression. This imbalance promotes angiogenesis, metastatic niche formation, and immune escape, and has been consistently associated with more aggressive disease, greater metastatic burden, and poorer survival across solid tumors (75). Our results are supported by the study of Wang et al. (76). Moreover, Thio et al. observed that higher NLR and PLR were both significantly and independently associated with worse survival in patients who were surgically treated for skeletal metastasis of the spine or long bone (27). A large meta-analysis identified elevated levels of NRL associated with poor survival also in several different cancer like lung, breast, endometrial cancer and multiple myeloma (77–79).

Based on the results of multivariate regression, a prognostic nomogram was constructed by including all variables (clinical and laboratory parameters) with a *p*-value < 0.05. The model provides a prediction of patient survival based on the total points obtained, making it a practical tool to assist clinicians in decision-making. By estimating expected survival, the nomogram can help guide personalized management, including the intensity of monitoring, selection of supportive care, and prioritization of therapeutic interventions tailored to the patient's prognosis and life expectancy. Furthermore, the design of the nomogram allows adaptation to other cancer-related BM cohorts, potentially supporting prognostic assessment across diverse tumor types. The resulting nomogram demonstrated strong predictive accuracy, particularly in the 12–24-month window, as evidenced by its ROC curves. Few studies adopt a pan-cancer approach and most focus on identifying risk factors for the development of BMs, rather than evaluating their prognostic significance (21). To our knowledge, no existing nomogram integrates prognostic biomarkers and the primary site in a pan-cancer cohort of surgically treated BM patients. Li et al. developed a nomogram aimed at predicting the prognosis of BM patients in an intensive care unit, to support risk stratification and treatment planning (43). Zhang et al. established a nomogram based only on serum bone metabolism and inflammatory biomarkers (neutrophils, platelets, CRP, osteocalcin, PINP, inflammatory burden index, NLR and β-CTX) to reflect OS (18). Interestingly, when comparing Zhang et al.'s results to ours, the AUCs of the ROC curves for 1-year and 2-year survival were comparable between the two models, while for 3-year survival, Zhang et al. (18) reported a slight better AUC (0.96 vs 0.74 in our study). Notably, Zhang et al.'s model did not account for the primary tumor type, highlighting the complementary nature of our pan-cancer approach that explicitly incorporates both tumor-specific and host-related factors for broader clinical applicability. Furthermore, some studies rely on biomarkers identified through genetic and molecular biology research, which are difficult to translate into clinical practice due to their limited accessibility and high cost (80, 81). Existing prognostic nomograms are focused on BMs from a single primary tumor. For example, Wang et al. found seven prognostic factors (tumor grade, histological type primary tumor size, tumor subtype, surgery, chemotherapy and number of metastatic organs) for BM breast cancer patients, which were used to construct a nomogram with achieving bootstrap-corrected C-indices of ~0.68–0.75 and 12- to 36-month AUCs up to 0.75 in both training and validation cohorts (82). Xu et al. (83) developed a nomogram using age, sex, race, tumor grade, T/N stages, local surgery, chemotherapy, radiotherapy, and brain or liver metastases as independent prognostic factors and found good predictive performance (AUC of 0.8 ca) at 3, 6 months and 1 year of follow-up.

Our pan-cancer nomogram synthesizes the strengths of these tumor-specific models by (i) incorporating both tumor type (breast, lung, renal, etc.) and universally measured biomarkers, and (ii) being developed in a prospective monocentric cohort of surgically treated bone-metastatic patients. This allows it to predict 3-years survival across a heterogeneous patient population.

These findings highlight the potential of integrating biomarker assessment into clinical practice to improve the prognostic stratification and management of patients with BMs.

This study has several limitations. First, it was conducted at a single center with a relatively small sample size, which may limit the statistical power and the external validity of the results. Second, although the inclusion of patients with BMs from various primary tumors (pan-cancer cohort) reflects real-world clinical complexity and may introduce biological heterogeneity that could confound the interpretation of biomarker associations, it also provides an opportunity to identify common features and prognostic markers across different tumor types. Third, the prognostic nomogram was not validated using an external independent cohort. Multi-center validation, ideally with larger and more diverse populations, will be crucial to confirm its generalizability. Moreover, it should be noted that all nomograms are tools that provide estimates but do not represent the full variability of survival prediction. This limitation also applies to our nomogram, which is therefore not a complete representation of the underlying model because it does not account for this variability. Finally, the absence of a control group of cancer patients without BM prevents direct comparison and limits the ability to draw causal inferences. Future studies should therefore include larger, multicenter cohorts with tumor-specific subgroups taking into account also for the different treatments and appropriate controls to validate and extend these findings.

## Conclusions

In summary, our prospective, monocentric cohort study demonstrates that a pan-cancer approach to BM can uncover both tumor-specific and cross-tumor prognostic biomarkers, ultimately enabling more nuanced risk stratification and could help in the personalized treatment planning. We identified significant variations in markers of bone turnover (BAP, P1NP), and systemic inflammation (NLR, RDW, LDH, IGFBP-3, HALP) across different primary tumor types, and showed that several of these—and particularly IGFBP-3, RDW, and NLR—retain independent prognostic value in the multivariate model. By integrating these clinical and laboratory parameters into a novel nomogram, we achieved good prediction of 12- to 24-month survival, with discrimination comparable to existing tumor-specific tools but offering the unique advantage of applicability across heterogeneous cohorts. Although further external validation is needed, our findings support the routine incorporation of selected biomarkers into the preoperative work-up of patients with BM and suggest that a pan-cancer model may help guide surgical and systemic therapeutic decisions in this complex population.

## Data Availability

Data sharing may be subject to institutional approval to ensure compliance with ethical regulations and participant confidentiality. The data supporting the findings of this study are available from the corresponding author upon reasonable request. Requests should include a brief description of the intended use.
