# Supplementary material for: Prognostic biomarkers for overall survival in patients undergoing surgery for bone metastases: a pan-cancer study
Source: Front Med (Lausanne). 2026 Jan 14;12:1655245. doi: 10.3389/fmed.2025.1655245 (PMC12847445; doi:10.3389/fmed.2025.1655245)
Supplement: Supplementary file 1 [file Data_Sheet_1.docx]

**Supplementary table 1: Reference Ranges for the analyzed biomarkers**

| **Marker** | **Normal range** |
| --- | --- |
| BAP, µg/L  Men  Women | 5.7 – 32,9  4.7 – 27.1 |
| IGFBP-3, µg/L | 1700 - 4000 |
| Beta-CTX, pg/mL |  |
| Premenopausal women  Postmenopausal women | 112.0 – 738.0  142.0 – 1351.0 |
| P1NP, µg/L | 28.0 – 128.0 |
| 1,25(OH)_2_D_3_, pmol/L | 47.8 – 190.3 |
| PTH, ng/L | 6.5 – 36.8 |
| Albumin, g/L | 35 - 52 |
| Osteocalcin, µg/L |  |
| Men  Premenopausal women  Postmenopausal women | 4.6 – 65.4  6.5 – 42.3  5.4 – 59.1 |
| Calcium, mmol/L | 2.10 – 2.55 |
| LDH, U/L | 135 - 214 |
| Beta 2 microglobulin, mg/L | 0.80 – 2.53 |
| CRP, mg/L | 0.00 – 5.00 |

BAP = Bone alkaline phosphatase; IGFBP-3 = Insulin-like growth factor binding protein-3; Beta-CTX-I = beta-isomerized C-terminal telopeptide of type I collagen; P1NP = total procollagen type 1 amino-terminal propeptide; 1,25(OH)2D = 1,25-dihydroxyvitamin D; PTH = Parathyroid hormone; LDH = Lactate DeHydrogenase; CRP = C-reactive protein.

**Supplementary table 2: Multivariate Cox proportional hazards model of bone markers.**

|  |  | | |
| --- | --- | --- | --- |
| **Predictors** | **Harzard Ratio** | **CI** | **p - value** |
| Sex [male] | 0.95 | 0.34 – 2.68 | 0.927 |
| Age | 1.04 | 1.00 – 1.08 | **0.042** |
| Site [humerus] | 1.21 | 0.40 – 3.64 | 0.737 |
| Site [rachis] | 0.79 | 0.10 – 6.06 | 0.822 |
| Site [other] | 1.71 | 0.23 – 12.71 | 0.600 |
| Surgery [prosthesis] | 0.74 | 0.30 – 1.80 | 0.503 |
| Surgery [other] | 0.97 | 0.22 – 4.34 | 0.969 |
| Primary_tumor [kidney] | 0.27 | 0.06 – 1.10 | 0.067 |
| Primary_tumor [lung] | 1.73 | 0.50 – 6.03 | 0.388 |
| Primary_tumor [other] | 0.45 | 0.15 – 1.34 | 0.151 |
| Calcium (0 = in range) | 1.93 | 0.70 – 5.31 | 0.200 |
| BAP (0 = in range) | 2.84 | 1.19 – 6.80 | **0.019** |
| IGFBP-3 (0 = in range) | 1.34 | 0.48 – 3.71 | 0.573 |
| Osteocalcin (0 = in range) | 1.19 | 0.36 – 3.89 | 0.777 |
| CTX (0 = in range) | 0.33 | 0.10 – 1.06 | 0.062 |
| P1NP (0 = in range) | 1.91 | 0.83 – 4.38 | 0.129 |
| Vitamin D (0 = in range) | 0.77 | 0.31 – 1.93 | 0.582 |
| PTH (0 = in range) | 1.27 | 0.57 – 2.82 | 0.560 |
| Observations | 53 | | |
| R^2^ | 0.303 | | |

BAP = Bone alkaline phosphatase; IGFBP-3 = Insulin-like growth factor binding protein-3; Beta-CTX-I = beta-isomerized C-terminal telopeptide of type I collagen; P1NP = total procollagen type 1 amino-terminal propeptide; 1,25(OH)2D = 1,25-dihydroxyvitamin D; PTH = Parathyroid hormone. Values with p < 0.05 are shown in bold.

**Supplementary Table 3: Multivariate Cox proportional hazards model of inflammatory and hematologic markers.**

|  |  | | |
| --- | --- | --- | --- |
| **Predictors** | **Estimates** | **CI** | **p - value** |
| Sex[male] | 0.50 | 0.14 – 1.73 | 0.272 |
| Age | 1.09 | 1.03 – 1.15 | **0.003** |
| Site [humerus] | 1.05 | 0.30 – 3.66 | 0.935 |
| Site [rachis] | 0.31 | 0.01 – 7.11 | 0.463 |
| Site [other] | 9.15 | 0.64 – 130.72 | 0.103 |
| Surgery [prosthesis] | 0.58 | 0.19 – 1.74 | 0.328 |
| Surgery [other] | 0.33 | 0.05 – 2.12 | 0.242 |
| primary_tumor [kidney] | 0.17 | 0.04 – 0.83 | **0.028** |
| primary_tumor [lung] | 1.19 | 0.39 – 3.93 | 0.772 |
| primary_tumor [other] | 0.47 | 0.14 – 1.63 | 0.234 |
| Albumin (0 = in range) | 2.91 | 0.69 – 12.25 | 0.145 |
| LDH (0 = in range) | 2.05 | 0.68 – 6.19 | 0.203 |
| β2-M (0 = in range) | 0.37 | 0.12 – 1.12 | 0.078 |
| CRP (0 = in range) | 1.61 | 0.40 – 6.51 | 0.506 |
| HGB (0 = in range) | 2.49 | 0.52 – 11.89 | 0.253 |
| RDW (0 = in range) | 2.73 | 1.07 – 6.94 | **0.035** |
| Neutrophils (0 = in range) | 0.87 | 0.24 – 3.13 | 0.829 |
| NLR | 1.06 | 1.00 – 1.21 | 0.053 |
| PLT (0 = in range) | 2.73 | 0.95 – 7.88 | 0.063 |
| PLR | 1.00 | 0.99 – 1.01 | 0.941 |
| SIRI | 0.95 | 0.78 – 1.14 | 0.558 |
| Monocytes (0 = in range) | 1.61 | 0.35 – 7.41 | 0.542 |
| WBC (0 = in range) | 1.09 | 0.36 – 3.27 | 0.883 |
| HALP | 1.00 | 0.90 – 1.12 | 0.942 |
| Observations | 54 | | |
| R^2^ | 0.426 | | |

LDH = Lactate DeHydrogenase; β2-M = beta 2 microglobulin; CRP = C-reactive protein; HGB=Hemoglobin; RDW= Red Blood Cell Volume distribution width; NLR= neutrophil-lymphocyte ratio; PLT=platelet; PLR= platelet-lymphocyte ratio; SIRI= systemic inflammation response index; WBC=white blood cells; HALP= Hemoglobin, Albumin, Lymphocyte, and Platelet counts (HALP). Values with p < 0.05 are shown in bold.
